# Polyimide-Derived Carbon-Coated Li_4_Ti_5_O_12_ as High-Rate Anode Materials for Lithium Ion Batteries

**DOI:** 10.3390/polym13111672

**Published:** 2021-05-21

**Authors:** Shih-Chieh Hsu, Tzu-Ten Huang, Yen-Ju Wu, Cheng-Zhang Lu, Huei Chu Weng, Jen-Hsien Huang, Cai-Wan Chang-Jian, Ting-Yu Liu

**Affiliations:** 1 Department of Chemical and Materials Engineering, Tamkang University, No. 151, Yingzhuan Road, Tamsui District, New Taipei City 25137, Taiwan; roysos@mail.tku.edu.tw; 2 National Synchrotron Radiation Research Center, 101 Hsin-Ann Road, Hsinchu Science Park, Hsinchu 30076, Taiwan; huang.ty@nsrrc.org.tw; 3 International Center for Young Scientists (ICYS), National Institute for Materials Science (NIMS), 1-2-1 Sengen, Tsukuba, Ibaraki 305-0047, Japan; WU.YenJu@nims.go.jp; 4 Material and Chemical Research Laboratories, Industrial Technology Research Institute, No. 195, Chung Hsing Road, Chutung, Hsinchu 31040, Taiwan; chengzhanglu@itri.org.tw; 5 Department of Mechanical Engineering, Chung Yuan Christian University, No. 200, Chungpei Road, Chungli District, Taoyuan City 32023, Taiwan; 6 Department of Green Material Technology, Green Technology Research Institute, CPC Corporation, No. 2, Zuonan Rd., Nanzi District, Kaohsiung City 81126, Taiwan; 295604@cpc.com.tw; 7 Department of Mechanical and Automation Engineering, I-Shou University, No.1, Sec. 1, Syuecheng Rd., Dashu District, Kaohsiung City 84001, Taiwan; 8 Department of Materials Engineering, Ming Chi University of Technology, 84 Gungjuan Road, Taishan District, New Taipei City 24301, Taiwan

**Keywords:** Li_4_Ti_5_O_12_, polyimide, carbon coating, lithium ion battery, rate performance

## Abstract

Carbon-coated Li_4_Ti_5_O_12_ (LTO) has been prepared using polyimide (PI) as a carbon source via the thermal imidization of polyamic acid (PAA) followed by a carbonization process. In this study, the PI with different structures based on pyromellitic dianhydride (PMDA), 4,4′-oxydianiline (ODA), and *p*-phenylenediamine (*p*-PDA) moieties have been synthesized. The effect of the PI structure on the electrochemical performance of the carbon-coated LTO has been investigated. The results indicate that the molecular arrangement of PI can be improved when the rigid *p*-PDA units are introduced into the PI backbone. The carbons derived from the *p*-PDA-based PI show a more regular graphite structure with fewer defects and higher conductivity. As a result, the carbon-coated LTO exhibits a better rate performance with a discharge capacity of 137.5 mAh/g at 20 C, which is almost 1.5 times larger than that of bare LTO (94.4 mAh/g).

## 1. Introduction

With the continuous depletion of fossil fuels and associated increasing air pollution, it is essential to raise the proportion of renewable energy supplies. However, renewable energy sources such as wind and solar are intermittent and cannot be stockpiled without energy storage systems (ESSs). ESSs based on lithium ion batteries (LIBs) are emerging as one of the key solutions to effectively integrate high shares of variable renewable energy due to their high energy density, zero memory effect and long lifespan [1]. By introducing an EES into the power generation system, it can smooth the output of wind or solar power generation, and reduce the impact on the power grid. Unfortunately, most of the anode and cathode materials suffer from low electronic conductivity and poor ionic diffusivity in the lattice resulting in poor rate capability. Therefore, the rate performance of the active materials must be further improved for use in voltage regulation and frequency modulation. 

To address the above issue, alien ion doping [2], the building of a nanoporous structure [3,4] and surface coating [5,6,7] have proven to be effective for improving the rate performance by narrowing the band gap, shortening migration paths for Li^+^ ions and reduction of interfacial resistance, respectively. Among these approaches, the surface coating is a feasible strategy to improve the LIB performance due to the multi-functional advantages such as enhancement of electric conductivity [8], structural stability [9] and offering a physical protection to avoid side reactions with the electrolyte [10]. Several compounds have been proposed to be an efficient coating layer such as carbon-based materials [11,12], metal oxides/hydroxides [13,14], phosphide-based materials [15] and glass-based materials [16]. Among these candidates, carbon coating is one of the most effective and facile ways to improve the electrochemical performance of LIB materials due to its excellent electron conductivity, low cost, and superior chemical/electrochemical stability. Compared with the metal oxide coating, carbon coating can easily form a smooth and uniform thin layer with high surface coverage on the active materials. Moreover, the carbon coating layer can also serve as a buffer layer to accommodate the dimensional variation of the active material during the lithiation and delithiation process leading to improved structural stability [17].

Recently, polyimide (PI) has been found to be a high-quality carbon source for the synthesis of graphene [18,19] and highly conductive carbon [20], because of the abundant hexagonal crystalline carbon within the imide structures. The obtained PI derived graphene reveals remarkable conductivity and electrochemical properties, which are useful for various applications e.g., supercapacitors [21], sensors [22], electrocatalysts [23] and electrothermal heaters [24]. Therefore, polyimide is expected to be suitable for forming the conductive carbon layer of LIB materials. It has been reported that the carbon films derived from PI with different structures can show quite different physical properties and graphite microstructure. However, the effect of carbon coating derived from different PIs on the LIB performance is still unclear.

In this study, two different PIs have been prepared from pyromellitic dianhydride (PMDA), 4,4′-oxydianiline (ODA) and *p*-phenylenediamine (*p*-PDA). By introducing the rigid planar moiety (*p*-PDA) into the main chain of classical PMDA/ODA PI (PO-PI), the PMDA/ODA/*p*-PDA PI (POP-PI) shows an increased crystallinity and orientation degree. Here, we used the PO-PI and POP-PI as carbon sources to modify the Li_4_Ti_5_O_12_ (LTO) anode material, followed by thermal treatment to obtain the carbon-coated LTO materials. The results indicate that the uniformly coated carbon layer on LTO can reduce its resistance and polarization leading to better rate performance and the corresponding electrochemical properties. Moreover, with incorporation of the rigid *p*-PDA segment, the carbon derived from the POP-PI exhibits better molecular packing and less defect. As a result, the carbon-coated LTO prepared from the POP-PI can display an even better kinetic performance than that obtained from the PO-PI.

## 2. Experimental Section

### 2.1. Material

The monomers ODA (97%), *p*-PDA (98%) and PMDA (97%) were purchased from Jinyu Co., Ltd. (Kaohsiung, Taiwan). The TiO_2_ (85%, anatase, Hombikat 8602) and Li_2_CO_3_ (≥99%, Aldrich) used for the synthesis of LTO were purchased from World Chem Industries Co., Ltd. (Taipei, Taiwan) and Sigma Aldrich (St. Louis, MI, USA), respectively.

### 2.2. Preparation of Pyromellitic Dianhydride/4,4′-Oxydianiline Polyimide (PMDA/ODA PI) and PMDA/ODA/p-Phenylenediamine (p-PDA) PI

In this study, the PI precursor, poly(amic acid) (PAA) was obtained through the two-steps synthesis method from its monomers. First, the ODA monomer or mixed ODA/*p*-PDA (1:1) were dissolved in *N*-methyl-2-pyrrolidone (NMP). Then, equimolar PMDA was added in the diamine solution under continuous stirring at 25 °C with a solid content of 15 wt% to produce the PAA solution. Here, the PAAs prepared from PMDA/ODA and PMDA/ODA/*p*-PDA are denoted as PO-PAA and POP-PAA, respectively. The PAA was then cast on glass substrate by doctor blade coating, s followed by soft baking at 80 °C for 60 min. The soft-baked precursor films were thermally imidized (150 °C for 30 min, 250 °C for 30 min, 350 °C for 60 min and 400 °C for 30 min) under N_2_ atmosphere to obtain the PMDA/ODA PI and PMDA/ODA/*p*-PDA PI denoted herein as PO-PI and POP-PI, respectively. 

### 2.3. Preparation of Carbon-Coated Li_4_Ti_5_O_12_ (LTO)

The carbon-coated LTO powders were prepared by spray drying precursor solution of lithium titanium peroxide, followed by solid-state calcination. Firstly, the TiO_2_ and Li_2_CO_3_ were dispersed in de-ionized water with a Li:Ti molar ratio 4:5 and a solid content of 15 wt%. The solution was ball milled for 12 h to produce the homogenous slurry, which was fed to a pilot spray dryer (OHKAWARA KAKOHKI, model L-8i, No. 145874). The spherical LTO precursor obtained was added to the PMDA/ODA or PMDA/ODA/*p*-PDA PAA solution, which was then stirred for 60 min and subsequently centrifuged. The centrifuged powders were dried in vacuum at 80 °C. Finally, the samples were thermally annealed via a stepwise process (150 °C for 30 min, 250 °C for 30 min, 350 °C for 60 min, 400 °C for 30 min, 500 °C for 60 min and 800 °C for 120 min). The modified LTO prepared from PO- and POP-PI are denoted herein as PO-LTO and POP-LTO, respectively. For the preparation of the bare LTO sample, the spray-dried precursor was directly annealed with the same heating program.

### 2.4. Characterization

The crystal structure of the sample was characterized by X-ray powder diffraction (XRD, Philips X’Pert/MPD instrument, El Dorado County, CA, USA). The morphologies were monitored by scanning electron microscopy (SEM, JEOL JSM-6701F, Tokyo, Japan) and transmission electron microscopy (TEM, JEOL 2010, Tokyo, Japan). The absorption measurement was taken using a Cintra 2020 (GBC scientific equipment, Australia) spectrophotometer. The differential scanning calorimetry (DSC) was measured on DSC 2500 (TA instrument, Lukens Drive, New Castle, DE, USA). Thermal gravimetric analysis (TGA) was carried out using a TGA 8000 (PerkinElmer, Boston, MS, USA). Functional group and chemical composition were characterized by using Fourier transform infrared (FTIR, PerkinElmer, Boston, MS, USA) spectroscopy and X-ray photoelectron spectroscopy (XPS, ULVAC-PHI, Tokyo, Japan). Raman spectrum of the as-prepared samples was measured using a Raman microscope (HR800, HORIBA, Tokyo, Japan). 

### 2.5. Electrochemical Analysis

The energy storage performance of the samples was evaluated by fabricating the coin cells in an argon filled glovebox. The working electrode was prepared by mixing active material, polyvinylidene fluoride (PVdF), KS4 and Super P with a ratio of 8:0.5:0.5:1 in *N*-methyl-2-pyrrolidone (NMP). The as-prepared slurry was cast onto a Al foil and then dried in a vacuum oven at 120 °C overnight. The electrolyte comprises 1.0 M LiPF_6_ dissolved in a mixture of ethylene carbonate and dimethyl carbonate at a volumetric ratio of 1:1. Cyclic voltammetry (CV) and electrochemical impedance spectroscopy (EIS) were carried out on a potentiostat (PARSTAT 4000 Potentiostat Galvanostat). The galvanostatic charge/discharge performance was examined between 1 and 2.5 V (vs. Li^+^/Li) at various C rates (1 C = 175 mA/g).

## 3. Results and Discussion

**Figure 1:**
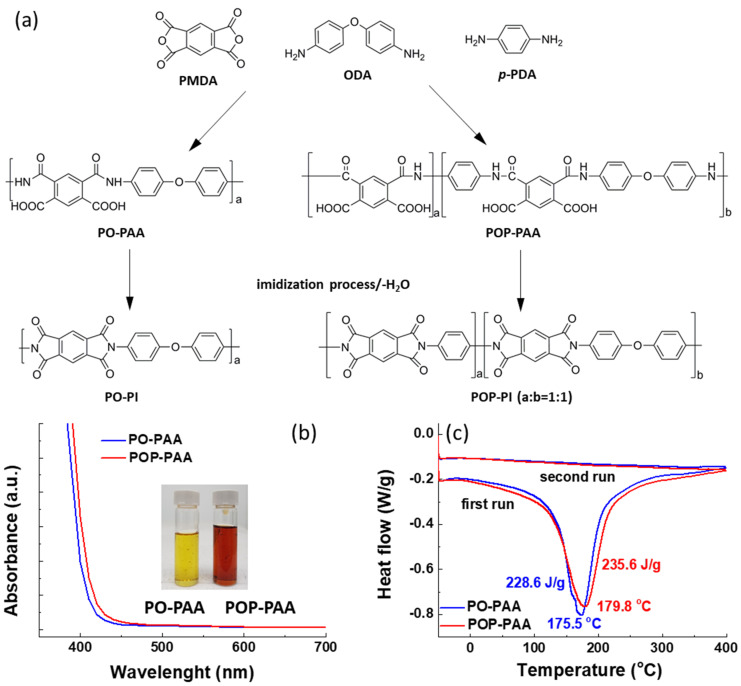
(**a**) The process of preparation of PMDA (pyromellitic dianhydride) and ODA (4,4′-oxydianiline) (PO-) and PMDA/ODA/*p*-phenylenediamine (*p*-PDA) polyimide (POP-PI) with PMDA, ODA, and *p*-PDA; (**b**) the absorbance spectrum of PO- and POP-PAA solution and (**c**) the differential scanning calorimetry (DSC) profile of the PO- and POP-PAA (poly(amic acid)).

In general, the molecular packing of PI can be improved by using rigid *p*-PDA units to replace the rotatable ODA moiety. It has been reported that the regular PI with higher orientation degree of molecular chains can produce better graphite structure after a carbonization process [25]. Therefore, in this study, we prepared the carbon-coated LTO powders using the less-ordered PO-PI and more ordered POP-PI as carbon sources. PO- and POP-PI were synthesized through a conventional two-step polycondensation of PMDA dianhydride with ODA and *p*-PDA diamines and the reaction process is shown in Figure 1a. The ultraviolet–visible (UV-vis) absorption spectra of the PO- and POP-PAA solutions are shown in Figure 1b. The inset in Figure 1b presents the digital photograph of the PO- and POP-PAA solutions. The PO-PAA solution exhibits strong absorption in the UV region with an absorption edge of 410 nm. With the incorporation of the PDA moiety into the polymer backbone, the absorption profile of POP-PAA tends to be shifted toward the long wavelength region. The red-shift of the absorption spectrum of POP-PAA can be explained by the formation of a charge transfer complex (CTC) between the alternating electron-acceptor (dianhydride) and electron-donor (diamine) moieties [26]. The C–O–C bond in ODA units can separate the chromaphoric centers and cut the electronic conjugations [27]. As a result, the PO-PAA shows a lighter color compared with that of POP-PAA due to its reduced intra-/intermolecular CTC formation. These results indicate that the PDA diamine units are successfully introduced into the copolymer. To study the effect of the *p*-PDA moiety on the imidization process, the enthalpy of imidization for the PO-PAA and POP-PAA was recorded by DSC measurement. As shown in Figure 1c, both the PAA samples exhibit a distinctive endothermic peak centered at around 175.5~179.8 °C in the first run which originated from the imidization reaction. In the second run, the DSC curves show a smooth profile without any peak, indicating the imidization process is complete. In addition, the enthalpy of imidization integrated from the DSC curve (first run) was calculated to be 228.6 and 235.6 J/g for PO-PAA and POP-PAA, respectively. The similar peak temperature and enthalpy of imidization for the two PAAs suggest that the incorporation of the *p*-PDA unit cannot alter the imidization behavior and the corresponding chemical conversion.

**Figure 2:**
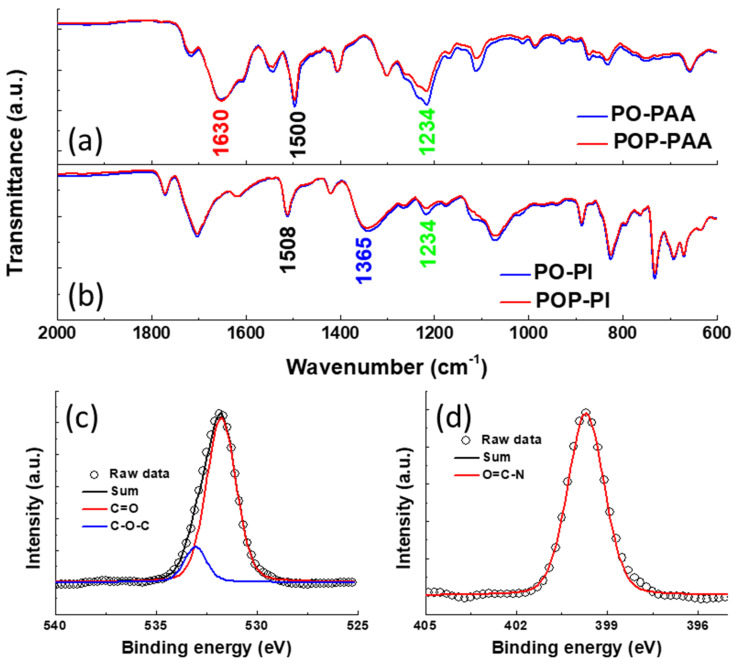
The Fourier transform infrared (FTIR) spectra of (**a**) PO-PAA and POP-PAA and (**b**) PO-PI and POP-PI; the peak deconvolution of the (**c**) O 1s and (**d**) N 1s X-ray photoelectron spectroscopy (XPS) spectra of the as-prepared PO-PI.

The FTIR spectra of the as-prepared PAA and its corresponding PI are shown in Figure 2a. Both the two PAAs exhibit a broad band located at 1630 cm^−1^, which can be assigned to amide C=O stretching mode [28,29]. After thermal treatment, this peak disappeared in the spectra of PO-PI and POP-PI (Figure 2b). Moreover, a new peak due to the C–N–C stretching vibration appears at 1365 cm^−1^ [30]. Meanwhile, the C=C double bond in the benzene ring at around 1500 cm^−1^ remains unchanged after the imidization process. These results indicate that the two PAAs were successfully converted into PO-PI and POP-PI after the imidization process. The peak at 1234 cm^−1^ is characteristic of C–O–C stretching vibration belonging to the ODA moiety [25]. It is worth noting that the POP-PAA and POP-PI reveal a much weaker intensity of C–O–C bond than their counterparts, confirming the successful incorporation of PDA moiety into the polymer backbone. The XPS O 1s and N 1s spectra of PO-PI are also shown in Figure 2c,d. After deconvolution, the O 1s XPS spectrum of the PO-PI can be fitted with two peak components assigned to C=O and C–O–C species at 531.9 and 533.2 eV, respectively [31]. For the N 1s spectrum of the PO-PI, only one characteristic peak contributed from O=C–N specie at 399.7 eV can be obtained after deconvolution [32]. These results indicate that the PAA has been successfully converted into the PI structure after the thermal treatment.

**Figure 3:**
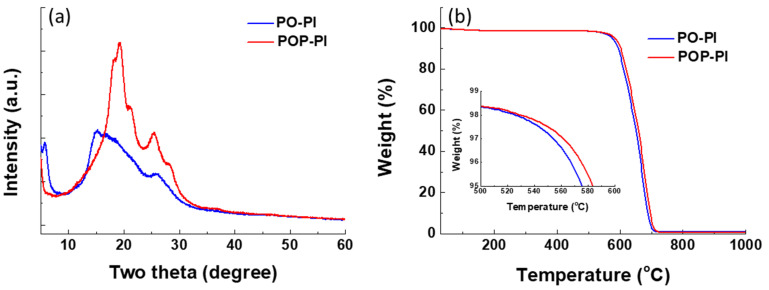
(**a**) The XRD patterns of PO-PI and POP-PI; (**b**) the TGA profile of the PO-PI and POP-PI.

The microscopic molecular packing state of the PO- and POP-PI films was investigated by XRD measurement. As shown in Figure 3a, the XRD pattern of PO-PI is blunt without obvious diffraction signals, suggesting its amorphous nature. The amorphous state of PO-PI originated from the flexible ether linkage within the ODA unit which loosens the chain packing of the PO-PI. In contrast, incorporation of the *p*-PDA unit in the polymer backbone can significantly enhance the polymer chain stacking. The regularly ordered structure of POP-PI is attributed to the rigid and planar skeletal structure of *p*-PDA leading to the better crystallinity [33]. Figure 3b compares the TGA curves of the PIs to investigate the thermal stability. Both the PI samples exhibit a major thermal decomposition ranging from 530 to 720 °C. The 5 wt% thermal decomposition temperature (*T*_d_) of the POP-PI is found to be 584 °C, which is higher than that of PO-PI (576 °C), suggesting its better thermal stability of POP-PI. The improved thermal stability of POP-PI can be interpreted by the presence of the *p*-PDA group, which increases the intra- and interpolymer chain interactions, resulting in tight polymer chain packaging. 

**Figure 4:**
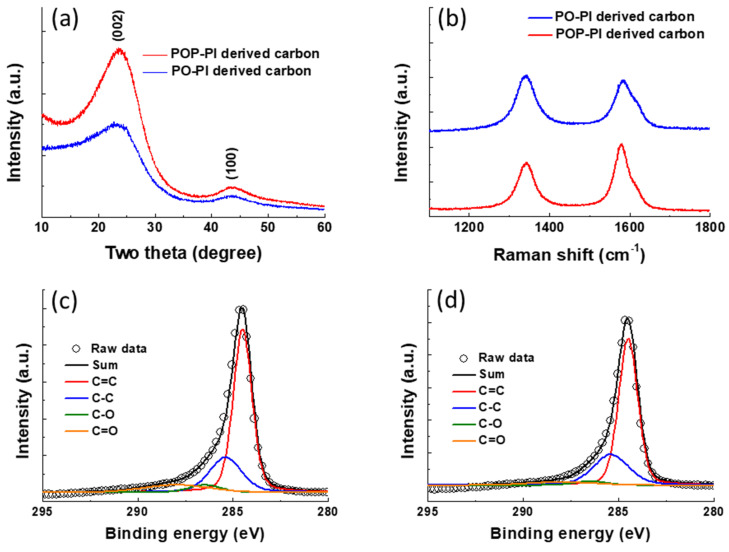
(**a**) The X-ray diffraction (XRD) pattern of the carbon materials derived from PO- and POP-PI; (**b**) Raman spectra of the carbon materials derived from PO- and POP-PI; peak deconvolution of the C 1s XPS spectra of (**c**) PO-PI derived carbon and (**d**) POP-PI derived carbon.

Figure 4a displays the XRD pattern of the carbon materials derived from PO- and POP-PI. The resultant carbon materials reveal two peaks located at 23.5 and 43.8°, indicating the development of (002) and (100) planes, respectively [34]. These results suggest that both the two PI samples can be converted into the hexagonal structures of the carbonized materials after the thermal treatment. Moreover, the POP-PI derived carbon exhibits much stronger XRD peaks than that derived from PO-PI, indicating its higher degree of graphitization. Figure 4b shows the Raman spectra of the two carbonized materials to further provide atomic-scale structural information. The Raman spectrum of the two samples shows the characteristic D band (1342 cm^−1^) and G band (1582 cm^−1^), which correspond to the sp^3^ and sp^2^ carbon, respectively [35,36]. In general, the intensity ratio between the D band and G band (I_D_/I_G_) can be used to determine the graphitization degree of the carbonized materials. The I_D_/I_G_ values of the PO- and POP-PI derived carbons are calculated to be 1.04 and 0.75, respectively. The lower I_D_/I_G_ ratio of POP-PI derived carbon also suggests its more ordered structure and the increased sp^2^ content. The C 1s XPS spectra of the PO- and POP-PI derived carbons are shown in Figure 4c,d to further characterize their chemical composition. Both the samples show four peaks with binding energies of 284.5, 285.4, 286.5 and 288.0 eV, which correspond to the functional groups of C=C, C–C, C–O and C=O, respectively. Based on the XPS analysis, the proportions of C=C for PO- and POP-PI derived carbons are 63.3% and 66.4%, respectively. Conversely, the proportion of oxygen-containing functional groups (C–O and C=O) of POP-PI based carbon (6.98%) is lower than that of the PO-PI based one (10.77%). In addition, the electronic conductivity of the PI derived carbons was directly measured with a four-point probe. The values of PO-PI and POP-PI derived carbons are 89.5 and 100.1 S/cm, respectively. These results further confirm that it is crucial to ensure the increased level of the structural arrangement of the initial PI precursor to prepare high-quality carbon materials with graphite-like structure [37].

**Figure 5:**
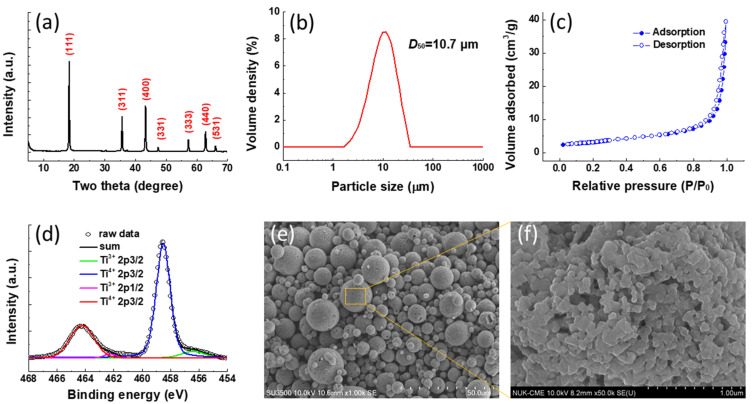
(**a**) The XRD pattern of the spray-dried Li_4_Ti_5_O_12_ (LTO) powder; (**b**) the particle size distribution of the LTO; (**c**) the nitrogen adsorption/desorption isotherms of the LTO; (**d**) peak deconvolution of the Ti 2p XPS spectrum of LTO powder; (**e**,**f**) scanning electron microscopy (SEM) images of the LTO with different magnifications.

The XRD pattern of the spray-dried LTO powders is exhibited in Figure 5a. All the peaks between 5 and 70° can be assigned to the spinel LTO structure without any impurity phases. The particle size distribution of the as-prepared LTO is shown in Figure 5b. The particle size of the LTO powders ranges between 1.88 and 27.4 μm with a mean particle size of 10.7 μm (*D*_50_). The micro-sized particles can be favorable for the powder packing during the electrode fabrication leading to better energy density. The surface area of the LTO was also evaluated by the Brunauer–Emmett–Teller (BET) analysis as shown in Figure 5c. Based on the nitrogen adsorption/desorption isotherms, the surface area of the LTO was calculated to be 11.3 m^2^/g. Figure 5d displays the Ti 2p core level XPS spectrum of the as-prepared LTO samples. There are two pairs of Ti 2p peaks observed at 464.3, 458.5 and 462.2, 456.4 eV for Ti^4+^ 2p_1/2_, Ti^4+^ 2p_3/2_ and Ti^3+^ 2p_1/2_, Ti^3+^ 2p_3/2_, respectively. The partial reduction of the Ti ions from Ti^4+^ to Ti^3+^ originates from the generation of oxygen vacancies during the thermal annealing in N_2_ ambience [38]. The microstructure of the LTO was investigated by SEM observation. The low-magnification SEM examination as shown in Figure 5e reveals that the morphology of LTO sample is perfectly preserved as highly uniform microspheres. In addition, the enlarged SEM image (Figure 5f) shows that the surface of the microspheres is composed of a primary nanoparticle with an average size of around 80 nm leading to a porous surface. The porous structure can facilitate the ionic transport during the charge/discharge process.

**Figure 6:**
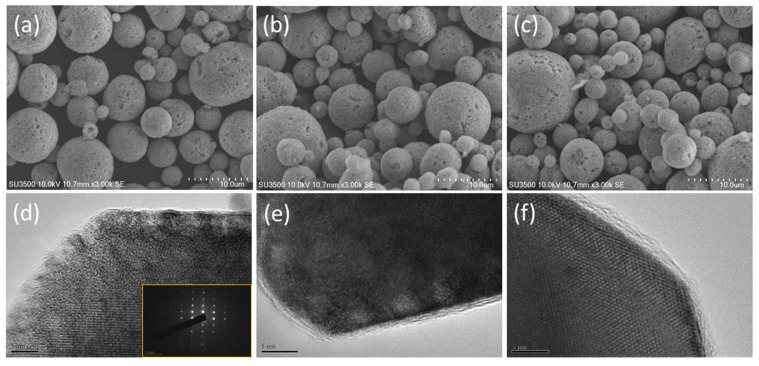
The SEM morphology of (**a**) bare LTO; (**b**) PO-LTO and (**c**) POP-LTO; the transmission electron microscopy (TEM) image of (**d**) bare LTO; (**e**) PO-LTO and (**f**) POP-LTO.

The morphologies of the pristine and carbon-coated LTO are monitored by SEM and TEM as shown in Figure 6. It can be observed that the whole sample reveals similar SEM morphology, indicating the spherical structure can be maintained after the carbon coating process. Moreover, the high-resolution TEM images of the PO-LTO and POP-LTO show a carbon layer with a thickness of around 2 nm was uniformly deposited on the LTO surface. In contrast, no carbon layer can be observed for bare LTO. It has been reported that the carbon layer can offer a conductive pathway for the electron transport. In addition, the randomly distributed defects and vacancies within the carbon layer also can improve the Li^+^ ion migration [6]. As a result, the kinetic balance between the electronic and ionic transport can be established leading to better rate performance. The XPS survey of the POP-LTO and Raman spectrum of the three samples are also provided in the Supporting Information.

**Figure 7:**
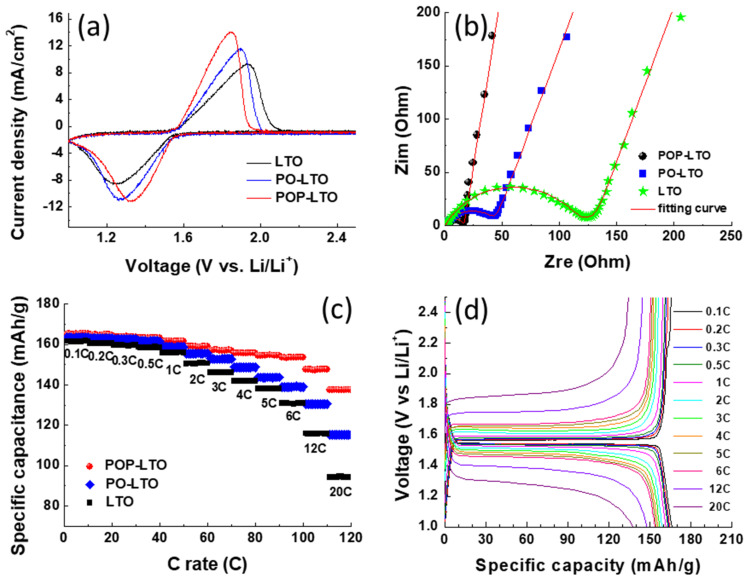
(**a**) The cyclic voltammetry (CV) curves of bare LTO, PO-LTO and POP-LTO stepped between 1.0 and 2.5 V with a scan rate of 1 mV/s; (**b**) Nyquist plots of bare LTO, PO-LTO and POP-LTO in the frequency range of 10^5^ Hz to 10^−2^ Hz; (**c**) rate capability of the as-prepared samples at C rates between 0.1 C and 20 C; (**d**) the corresponding charge/discharge profile of the POP-LTO with various C rates.

The electrochemical properties of the bare and carbon-coated LTO samples were studied by measuring their CV profiles with a scan rate of 1 mV/s between 1.0 and 2.5 V as shown in Figure 7a. All the samples reveal a pair of sharp redox couple which corresponds to the transition between Li_4_Ti_5_O_12_ and Li_7_Ti_5_O_12_. The separation between the anodic and cathodic peaks (∆E) of the LTO would be dramatically reduced with the incorporation of carbon coating, indicating the reduction of polarization. The ∆E of LTO, PO-LTO and POP-LTO is found to be 0.7, 0.63 and 0.52 V, respectively. The lower polarization is mainly attributed to the enhancement of electric conductivity by the interfacial carbon modification. As shown in Figure 4, the POP based carbon layer shows a more regular graphite structure with a higher conductivity (100.1 S/cm) than that of PO based carbon layer (89.5 S/cm). As a result, the POP-LTO reveals a much lower polarization (0.52 V) due than that of PO-LTO (0.63 V). Furthermore, the EIS spectra of the three electrodes were taken to investigate the interfacial impedance in the electrodes. Figure 7b shows the Nyquist plots of LTO, PO-LTO and POP-LTO electrodes with a frequency range from 10^5^ Hz to 10^−2^ Hz at an amplitude of 10 mV. All the plots exhibit a semicircle with a sloping line. The charge transfer resistance (*R*ct) determined from the size of the semicircle in the high frequency is around 117, 43.7 and 21.5 Ω for LTO, PO-LTO and POP-LTO, respectively. The *R*ct of POP-LTO is much lower than that of the bare one due to the deposition of the conductive carbon layer. The rate performance of the three electrodes evaluated at various C rates is displayed in Figure 7c. As expected, the POP-LTO with the modification of the POP-PI derived carbon layer can deliver the best rate capability due to its low polarization and *R*ct. The POP-LTO shows a high capacity retention of 83.2% (137.5 mAh/g) at a high rate of 20 C, which is higher than 70.7% (115.3 mAh/g) and 58.5% (94.4 mAh/g) for PO-LTO and pristine LTO, respectively. The corresponding charge/discharge profiles of the POP-LTO with different C rates are also provided in Figure 7d. The comparison of LTO performance with different carbon coating is also summarized in the Supporting Information.

## 4. Conclusions

In summary, a facile and scalable method has been developed for synthesizing carbon-coated LTO, using PI as the carbon source. After carbonization treatment at 800 °C in N_2_ ambience, the PI coating can be transferred into the high-conductive carbon layer. The nano-carbon layer on LTO can improve both the electronic and ionic conductivities. As compared to pristine LTO, results show that the POP-LTO exhibits substantially improved cell performances particularly in the rate capability. A high initial discharge capacity of 165.1 mAh/g is delivered at 0.1 C and the specific capacity still maintain 137.5 mAh/g even at 20 C. It is believed that the developed method also can be extended to improve the electrochemical performances of other alternative LIB materials with low intrinsic electrical conductivity and poor Li^+^ diffusion coefficient.

## Supplementary Materials

The following are available online at https://www.mdpi.com/article/10.3390/polym13111672/s1, Figure S1: The XPS survey of the POP-LTO and the corresponding atomic percentages, Figure S2: Raman spectrum of the pristine LTO, PO-LTO and POP-LTO, Table S1: The comparison of LTO performance with different carbon coating.
