# Supplementary material for: Polyimide-Derived Carbon-Coated Li4Ti5O12 as High-Rate Anode Materials for Lithium Ion Batteries
Source: Polymers (Basel). 2021 May 21;13(11):1672. doi: 10.3390/polym13111672 (PMC8196661; doi:10.3390/polym13111672)
Supplement: Supplementary file 1 [file polymers-13-01672-s001.zip › polymers-1215866-supplementary.pdf]

Supporting Information

Polyimide-Derived Carbon-Coated $Li_4Ti_5O_{12}$ as High-Rate Anode Materials for Lithium Ion Batteries

Shih-Chieh Hsu[1], Tzu-Ten Huang,[2] Yen-Ju Wu,[3] Cheng-Zhang Lu,[4] Huei Chu Weng,[5,*]

Jen-Hsien Huang,[6] Cai-Wan Chang-Jian[7,*] and Ting-Yu Liu[8,*]

[1]Department of Chemical and Materials Engineering, Tamkang University, No. 151, Yingzhuan Road, Tamsui District, New Taipei City, 25137, Taiwan

[2]National Synchrotron Radiation Research Center, 101 Hsin-Ann Road, Hsinchu Science Park, Hsinchu 30076, Taiwan

[3]International Center for Young Scientists (ICYS), National Institute for Materials Science (NIMS), 1-2-1 Sengen, Tsukuba, Ibaraki 305-0047, Japan

[4]Material and Chemical Research Laboratories, Industrial Technology Research Institute, No. 195, Chung Hsing Road, Chutung, Hsinchu 31040, Taiwan

[5]Department of Mechanical Engineering, Chung Yuan Christian University, No. 200, Chungpei Road, Chungli District, Taoyuan City 32023, Taiwan

[6]Department of Green Material Technology, Green Technology Research Institute, CPC Corporation, No.2, Zuonan Rd., Nanzi District, Kaohsiung City, 81126, Taiwan

[7]Department of Mechanical and Automation Engineering, I-Shou University, No.1, Sec. 1, Syuecheng Rd., Dashu District, Kaohsiung City 84001, Taiwan

[8]Department of Materials Engineering, Ming Chi University of Technology, 84 Gungjuan Road, Taishan District, New Taipei City, 24301, Taiwan, ROC

The XPS survey and the corresponding atomic percentages of the POP-LTO are shown in Fig. S1. It should be noted that the effective depth of the XPS measurement is approximately 5 nm. Therefore, the atomic percentages is not a typical value for the overall sample. The Raman spectra of pristine and carbon-coated LTO are shown in Fig. S2. The five Raman bands located at 237, 339, 430, 675 and 760 cm^{-1} are the feature of the spinel structure (A_{1g}+E_g+3F_{2u}) [1]. The results indicate that the carbon coating cannot change the atomic arrangement of LTO. The comparison of LTO performance with different carbon coating is also summarized in Table S1.

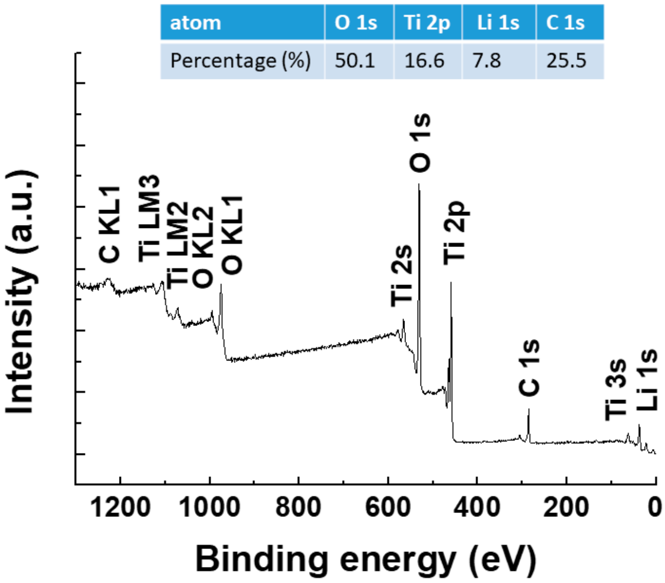

Figure S1. The XPS survey of the POP-LTO and the corresponding atomic percentages.

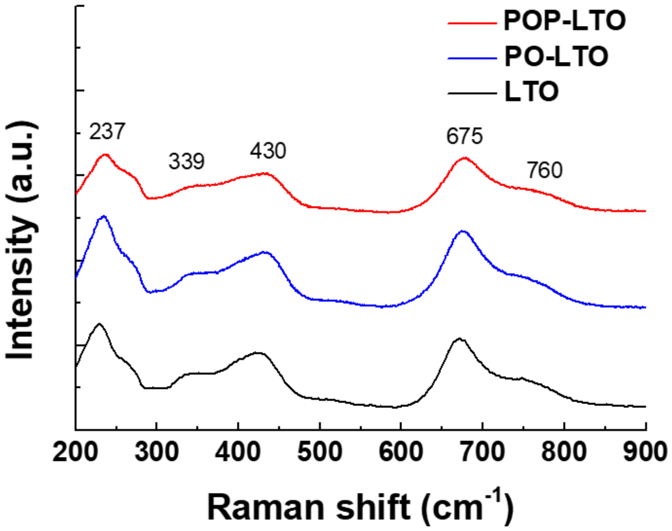

Figure S2. Raman spectrum of the pristine LTO, PO-LTO and POP-LTO.

Table S1. The comparison of LTO performance with different carbon coating

Carbon Source	Capacity (mAh/g)	Capacity (mAh/g)	Ref.
glucose	168 at 0.1 C	60 at 10 C	[2]
glucose	170 at 0.2 C	120 at 10 C	[3]
glucose	150 at 0.1 C	79.6 at 10 C	[4]
ethylenediamine	173.8 at 0.1 C	158 at 10 C	[5]
pitch	152.7 at 0.5 C	80 at 5 C	[6]
polyimide	165.1 at 0.1 C	137.5 at 20 C	This work

Reference

[1] Mukai, K.; Kato, Y.; Nakano, H. Understanding the zero-strain lithium insertion scheme of $Li[Li_{1/3}Ti_{5/3}]O_4$: Structural changes at atomic scale clarified by Raman spectroscopy. J. Phys. Chem. C **2014**, *118*, 2992−2999.

[2] Cheng, Q.; Tang, S.; Liang, J.; Zhao, J.; Lan, Q.; Liu, C.; Cao, Y.C. High rate performance of the carbon encapsulated $Li_4Ti_5O_{12}$ for lithium ion battery. Results in Physics **2017**, *7*, 810-812.

[3] Zhu, Z.; Cheng, F.; Chen, J. Investigation of effects of carbon coating on the electrochemical performance of $Li_4Ti_5O_{12}$/C nanocomposites. J. Mater. Chem. A **2013**, *1*, 9484-9490.

[4] Wang, P.; Zhang, G.; Cheng, J.; You, Y.; Li, Y.K.; Ding, C.; Gu, J.J.; Zheng, X.S.; Zhang, C.F.; Cao, F.F. Facile synthesis of carbon-coated spinel Li_4Ti5O_{12}/rutile TiO_2 composites as an improved anode material in full lithium-ion batteries with $LiFePO_4$@N-doped carbon cathode. ACS Appl. Mater. Interfaces **2017**, *9*, 7, 6138–6143.

[5] Long, D.H; Jeong, M.G.; Lee, Y.S.; Choi, W.; Lee, J.K.; Oh, I.H.; Jung, H.G. Coating Lithium Titanate with Nitrogen-Doped Carbon by Simple Refluxing for High-Power Lithium-Ion Batteries. ACS Appl. Mater. Interfaces **2015**, *7*, 19, 10250–10257.

[6] Gong, L.; Chen, Y.; Yu, H.; Liu, H.; Li, C.; Liu, Z.Q. Carbon-coated $Li_4Ti_5O_{12}$ anode materials synthesized using H_2TiO_3 as Ti source. J. Mater. Sci. Technol. **2014**, *30*, 1092-1095.
